# Supplementary material for: Wnt/β-catenin signaling regulates amino acid metabolism through the suppression of CEBPA and FOXA1 in liver cancer cells
Source: Commun Biol. 2024 Apr 29;7:510. doi: 10.1038/s42003-024-06202-9 (PMC11058205; doi:10.1038/s42003-024-06202-9)
Supplement: Supplementary file 2 — Supplementary Information [file 42003_2024_6202_MOESM2_ESM.pdf]

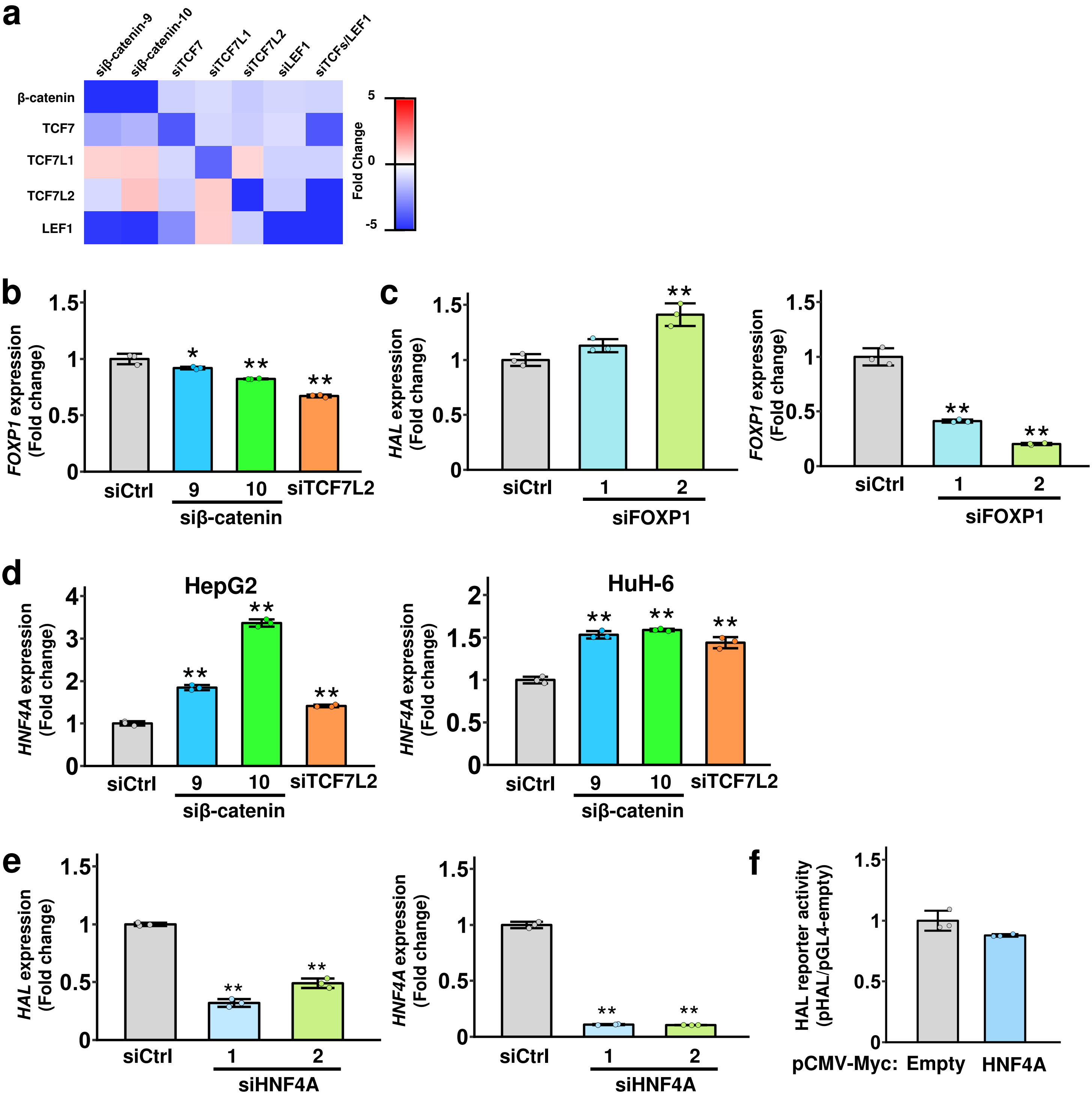

Supplementary Figure 1. The effects of FOXP1 and HNF4A on the expression and reporter activity of *HAL*

a The expression levels of β-catenin and TCF/LEF genes in response to the indicated siRNA are depicted in the heatmap. **b** The effect of β-catenin or TCF7L2 siRNA on the expression of *FOXP1* in HepG2 cells. The expression levels were assessed by RT-qPCR. **c** The expression of *HAL* and *FOXP1* in HuH-7 cells treated with control or two-independent FOXP1 siRNAs. The expression levels were measured by RT-qPCR. **d** Knockdown of β-catenin or TCF7L2 increased the expression levels of *HNF4A* in HepG2 and HuH-6 cells. **e** Suppressed expression of *HAL* by two-independent siRNAs against HNF4A in HuH-7 cells. The expression levels were assessed by RT-qPCR. **f** The effect of HNF4A on the reporter activity of pHAL-90/+147. Plasmid expressing HNF4A or the empty plasmid was co-transfected with the reporter plasmids in HepG2 cells. Unless specified otherwise, data are represented as the mean ± SD of three independent experiments. Statistical significance was determined by Dunnett's test (**b, c, d, e, and f**). *P<0.05, **P<0.01 vs siCtrl.

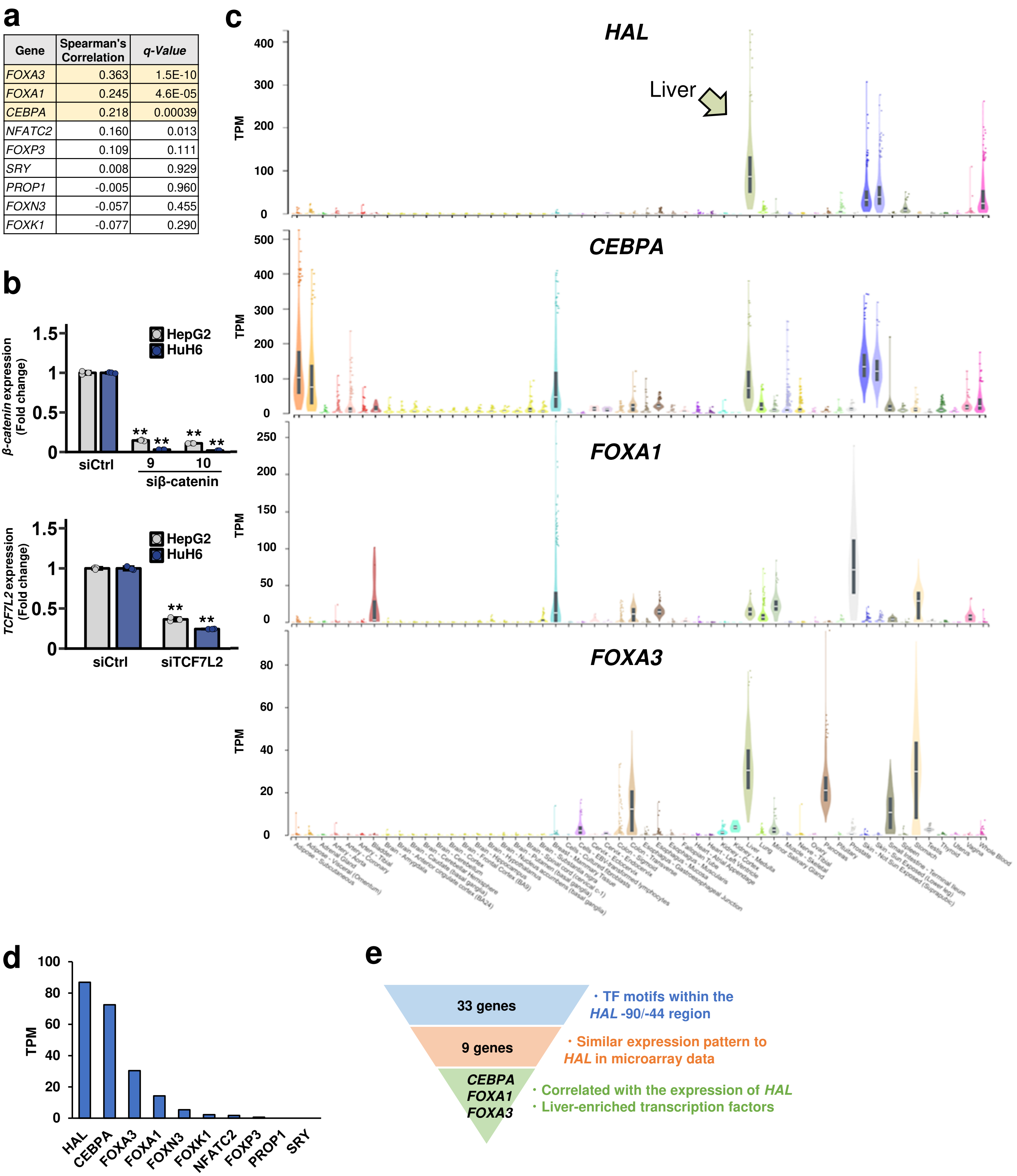

Supplementary Figure 2. Involvement of CEBPA, FOXA1, and FOXA3 in the regulation of HAL

a Correlation of *HAL* expression with that of nine transcription factors in hepatocellular carcinoma. Spearman's correlation of listed genes was analyzed using a hepatocellular carcinoma dataset (TCGA, Pan-Cancer Atlas). **b** Knockdown efficiency of β-catenin and TCF7L2 siRNAs was evaluated by RT-qPCR. Statistical significance was determined by Dunnett's test or unpaired two-tailed Student's t-test. ***P*<0.01 vs siCtrl. Data are represented as the mean ± SD of three independent experiments. **c** The expression of *HAL*, *CEBPA*, *FOXA1*, and *FOXA3* in normal tissues. The data were obtained from the GTEx Portal (https://gtexportal.org/home/). **d** The expression of *HAL* and the nine candidate TFs in normal liver tissue. The data were obtained from the GTEx Portal. **e** A flowchart of the strategy for the selection of candidate TFs.

a

TF name	Score	Start	End	Strand	Predicted sequence	Motif name
CEBPA	12.32	-70	-83	-	TAGTTGCTCAATAC	CBE-1
FOXA3	10.59	-50	-60	-	GAATAAACAAA	FBE
FOXA1	9.05	-49	-60	-	GAATAAACAAAT	FBE
CEBPA	8.70	-55	-68	+	TTATTCCACAAACA	CBE-2

b

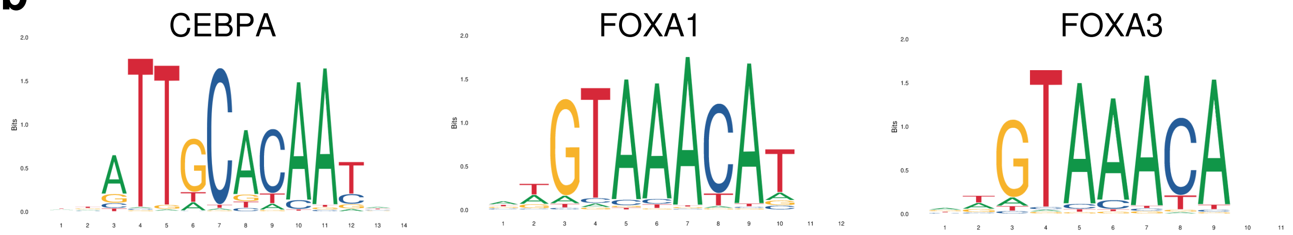

Supplementary Figure 3. Identification of CEBPA-, FOXA1-, and FOXA3-binding motifs in the promoter region (between -90 and -44) of *HAL*

a The JASPAR scores of TF-binding motifs between -90 and -44 in the *HAL* promoter region. The motifs with score greater than 8.0 are shown in the list. **b** DNA-binding motifs of CEBPA, FOXA1, and FOXA3 obtained from JASPAR database.

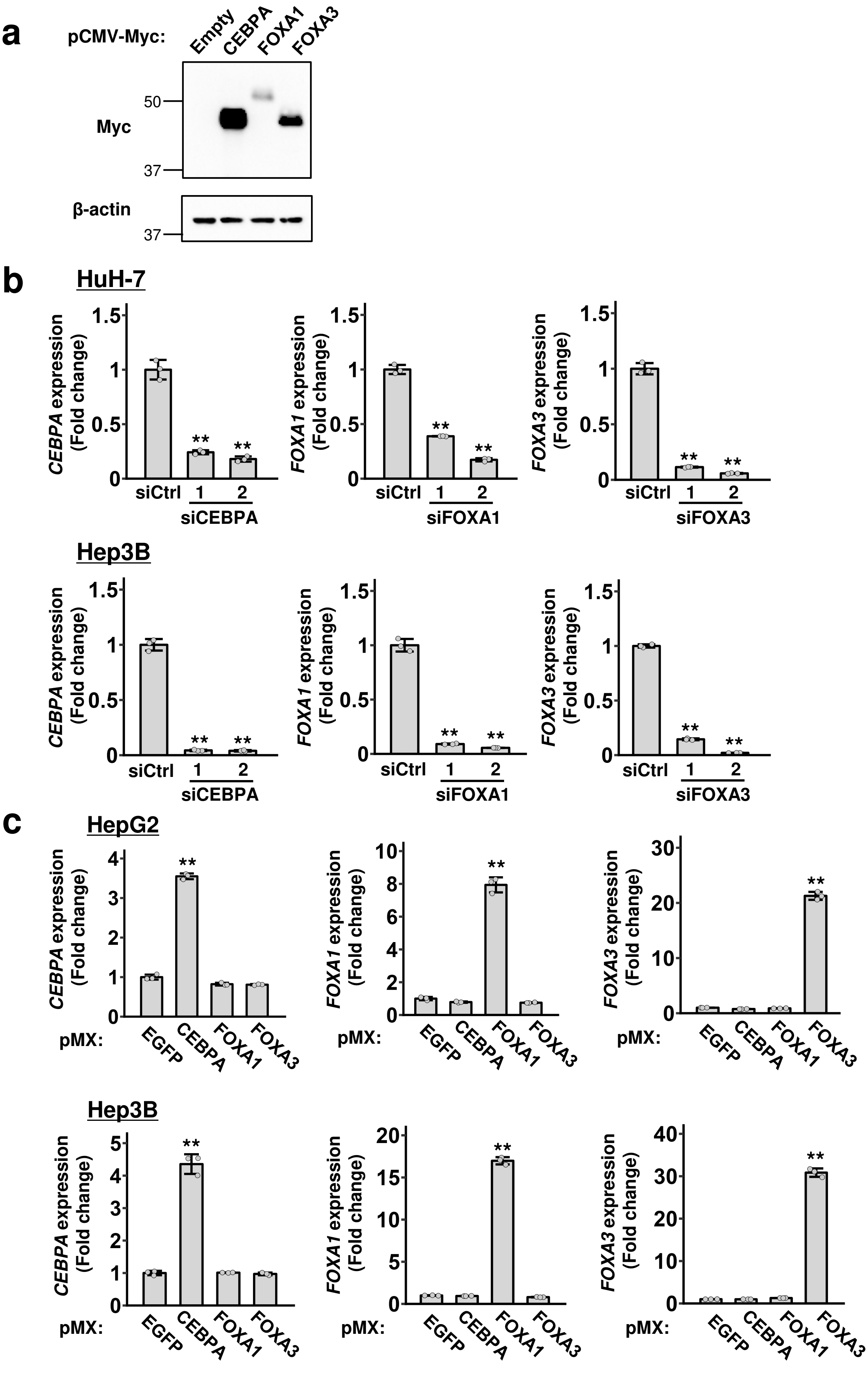

Supplementary Figure 4. The effect of indicated siRNAs or plasmid expressing *CEBPA*, *FOXA1*, or *FOXA3* on their expression in the liver cancer cells

a Overexpression of CEBPA, FOXA1, or FOXA3 in HuH-7 cells. Lysates from the cells transfected with plasmids of pCMV-Myc CEBPA, FOXA1, or FOXA3, were subjected to immunoblot analysis with anti-Myc antibody. **b** Knockdown efficiency of *CEBPA*, *FOXA1*, or *FOXA3* siRNA on their expression in HuH-7 and Hep3B cells. Their expression was evaluated by RT-qPCR. **c** The effect of retrovirus expressing *CEBPA*, *FOXA1*, or *FOXA3* on their expression in HepG2 and Hep3B cells. Unless specified otherwise, data are represented as the mean ± SD of three independent experiments. Statistical significance was determined by Dunnett's test **(b and c)**. $^{**}P$<0.01 vs siCtrl.

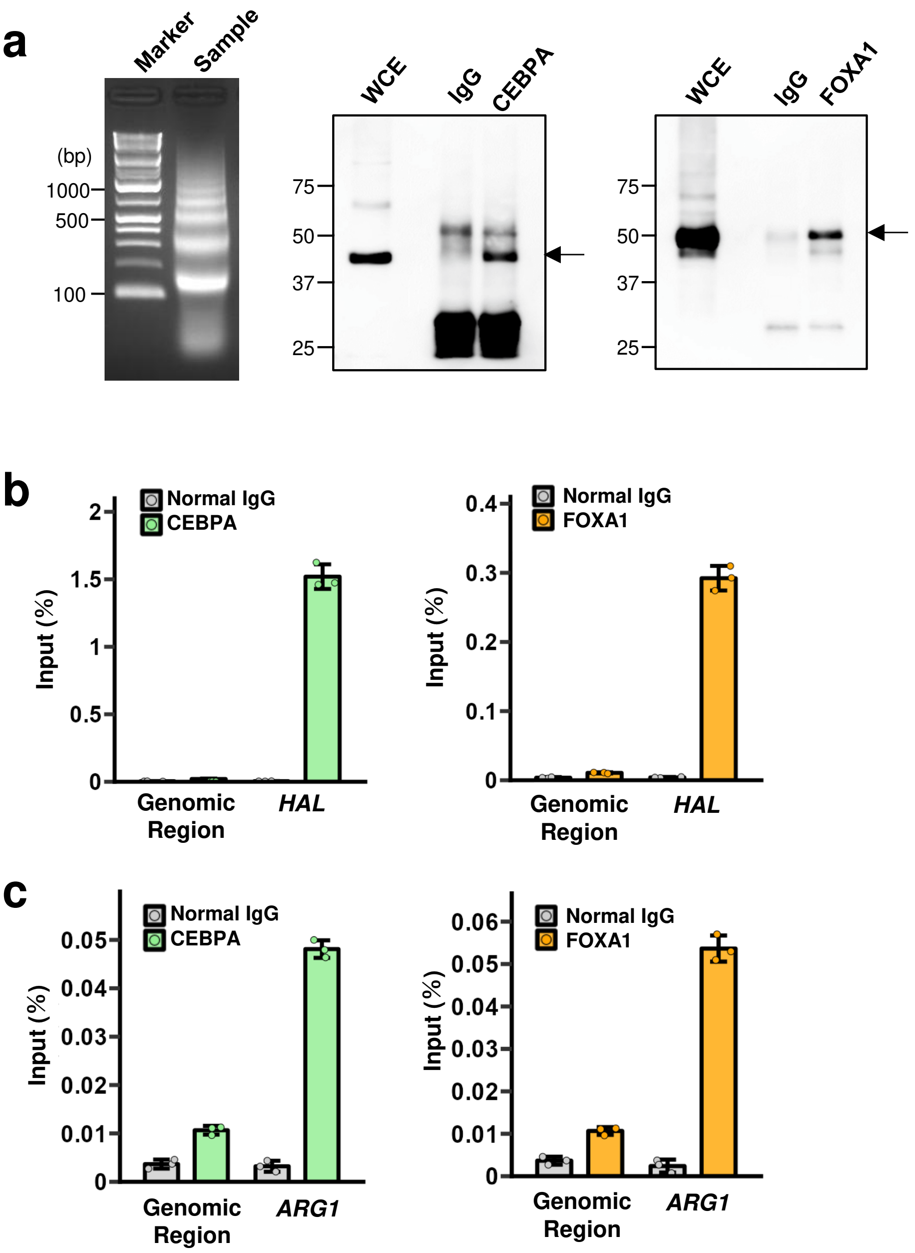

Supplementary Figure 5. Regulation of ARG1 by the Wnt/β-catenin signaling pathway via CEBPA and FOXA1
a Fragmentation of DNA extracted from HuH-7 cells for ChIP-seq (left). Efficacy of immunoprecipitation of CEBPA and FOXA1 was confirmed by immunoblot analysis with specific-antibodies (middle, anti-CEBPA antibody; right, anti-FOXA1 antibody). **b, c** Enrichment of the promoter regions of *HAL* (**b**) or intron1 of *ARG1* (**c**) was verified by ChIP-qPCR with anti-CEBPA or anti-FOXA1 antibody. Normal rabbit IgG was used as a negative control.

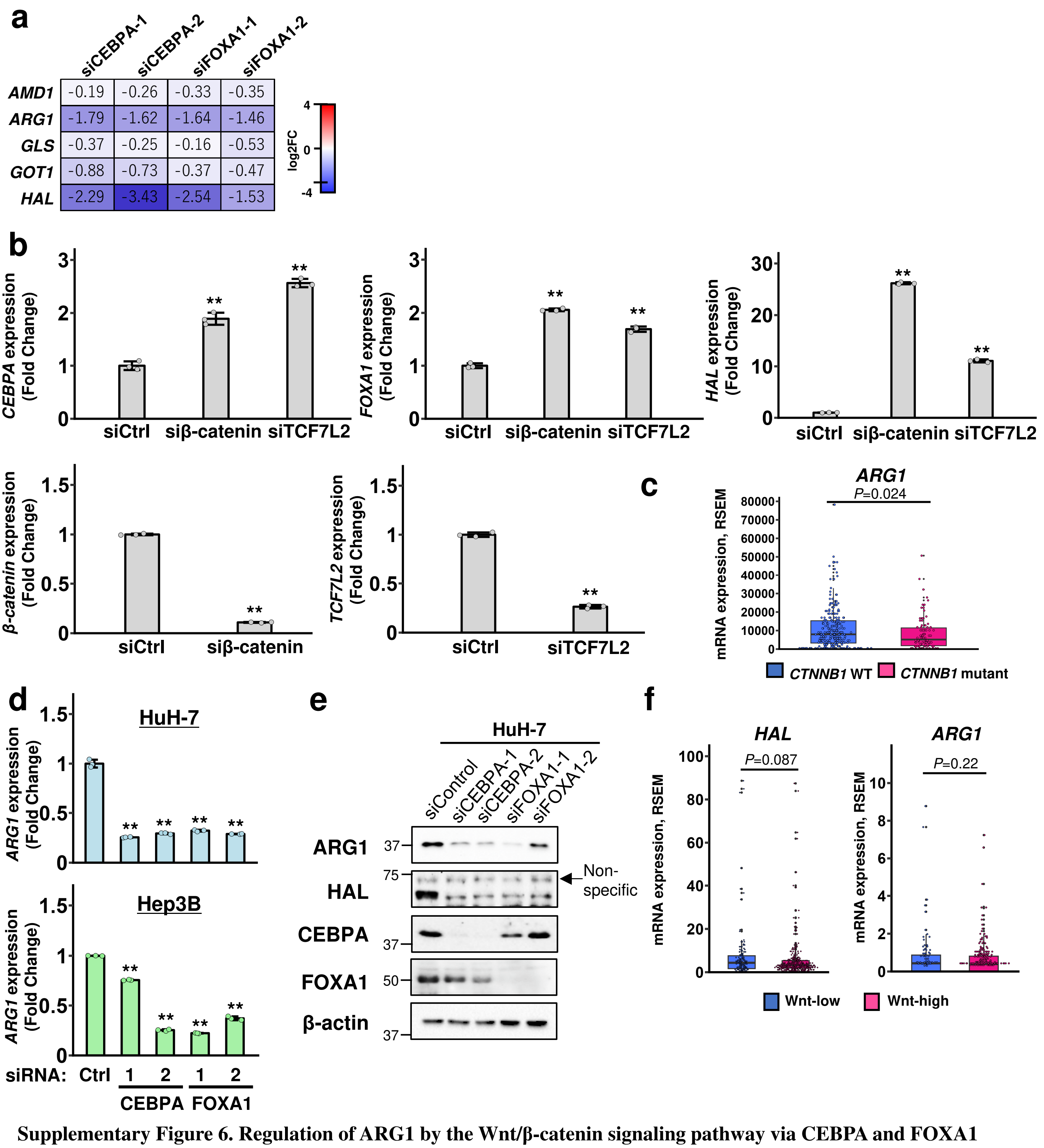

Supplementary Figure 6. Regulation of ARG1 by the Wnt/β-catenin signaling pathway via CEBPA and FOXA1
a Suppressed expression of *AMD1*, *ARG1*, *GLS*, *GOT1*, and *HAL* in response to CEBPA or FOXA1 siRNAs in HuH-7 cells. The values represent log2 fold change (FC) of their expression in response to CEBPA or FOXA1 siRNA compared to control siRNA. **b** The effect of β-catenin or TCF7L2 siRNA on the expression of *CEBPA*, *FOXA1*, *HAL*, *β-catenin*, and *TCF7L2* in HepG2 cells. Their expression levels were assessed by RT-qPCR. *HPRT1* was used as an internal control. The data represent mean ± SD from three-independent experiments. Statistical significance was determined by unpaired two-tailed Student's t-test or Dunnett's test. ***P*<0.01 vs siCtrl. **c** Association between the expression of *ARG1* and *CTNNB1* mutation status. The data were obtained from a dataset comprised of 361 hepatocellular carcinomas (TCGA, Pan-Cancer Atlas). Statistical significance was determined by unpaired two-tailed t-test. Center line, median; Box limits, upper and lower quartiles; Whiskers, 1.5x interquartile range. **d** Two-independent CEBPA or FOXA1 siRNAs markedly reduced *ARG1* expression in HuH-7 and Hep3B cells. Their expression was assessed by RT-qPCR. **e** The decreased expression of ARG1 and HAL was confirmed by immunoblotting. **f** No association between the expression levels of *HAL* (532 samples) or *ARG1* (359 samples) and Wnt-status was observed in colorectal cancer tissues (TCGA, Pan-Cancer Atlas). Wnt-high: colorectal cancer tissues with pathological mutations in *APC*. Wnt-low: colorectal cancer tissues with non-pathological mutations in *APC*. Statistical significance was determined by unpaired two-tailed t-test. Center line, median; Box limits, upper and lower quartiles; Whiskers, 1.5x interquartile range.

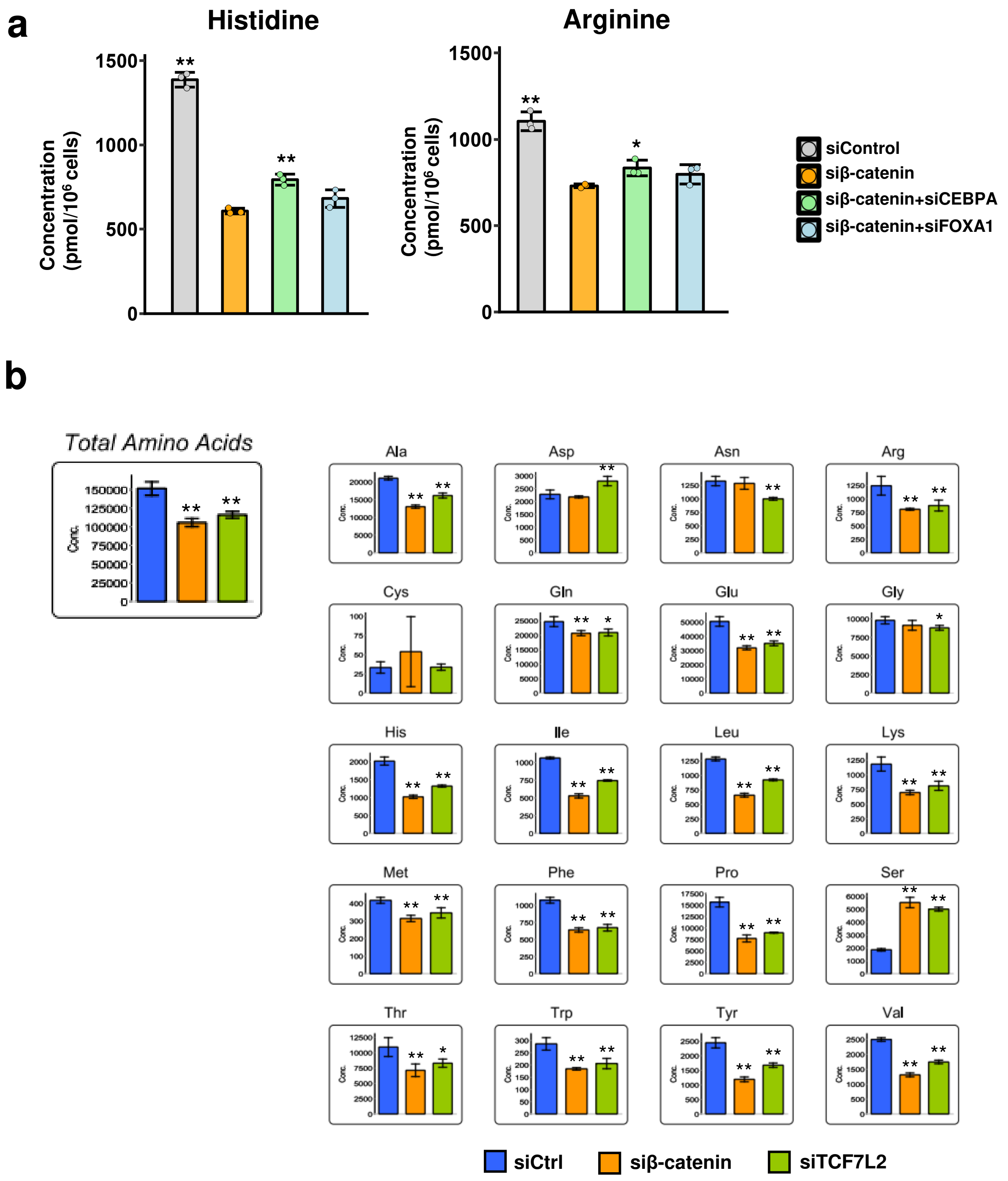

Supplementary Figure 7. Change in the levels of amino acids in liver cancer cells

a Quantitative analysis of the indicated metabolites using mass spectrometry in the HepG2 cells treated with the indicated siRNAs. Statistical significance was determined by Dunnett's test. **P*<0.05, ***P*<0.01 vs siβ-catenin. **b** Levels of amino acids in HepG2 cells treated with control, β-catenin, or TCF7L2 siRNA. The data represent mean ± SD from three-independent cultures. Statistical significance was determined by Dunnett's test. **P*<0.05, ***P*<0.01 vs siCtrl.

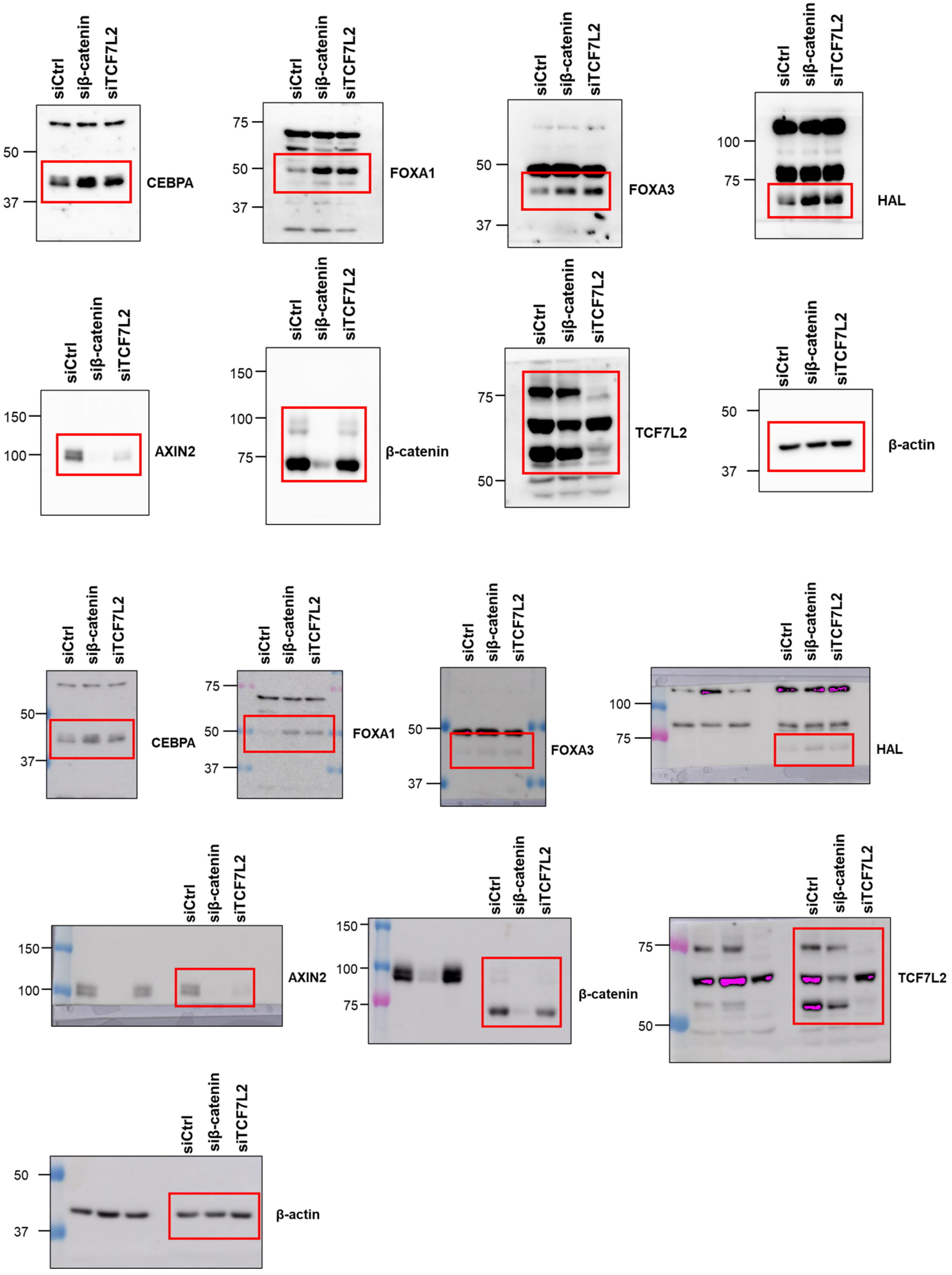

Supplementary Figure 8. Uncropped and unedited blot images with/without ladder of Figure 1c

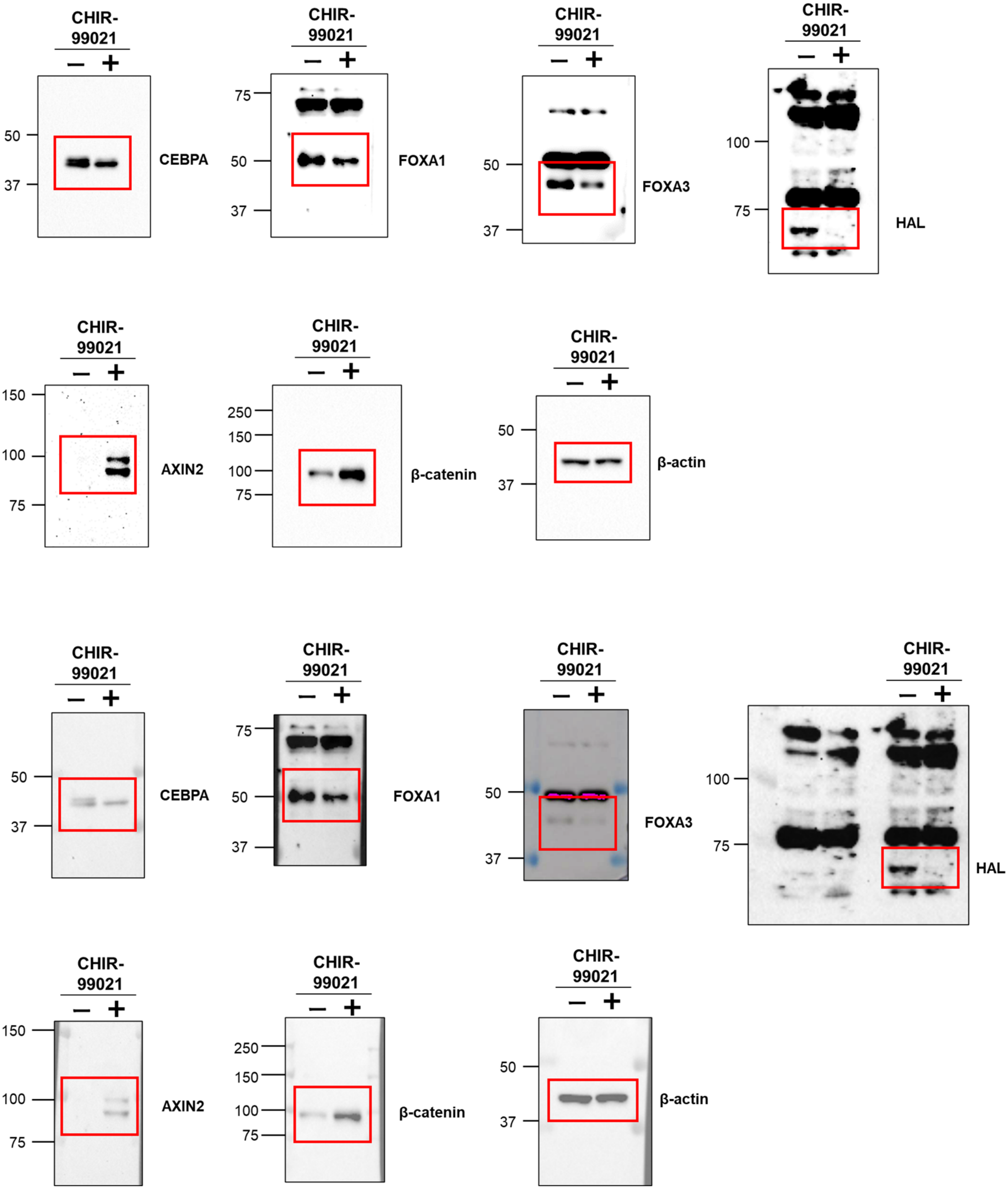

Supplementary Figure 9. Uncropped and unedited blot images with/without ladder of Figure 1d

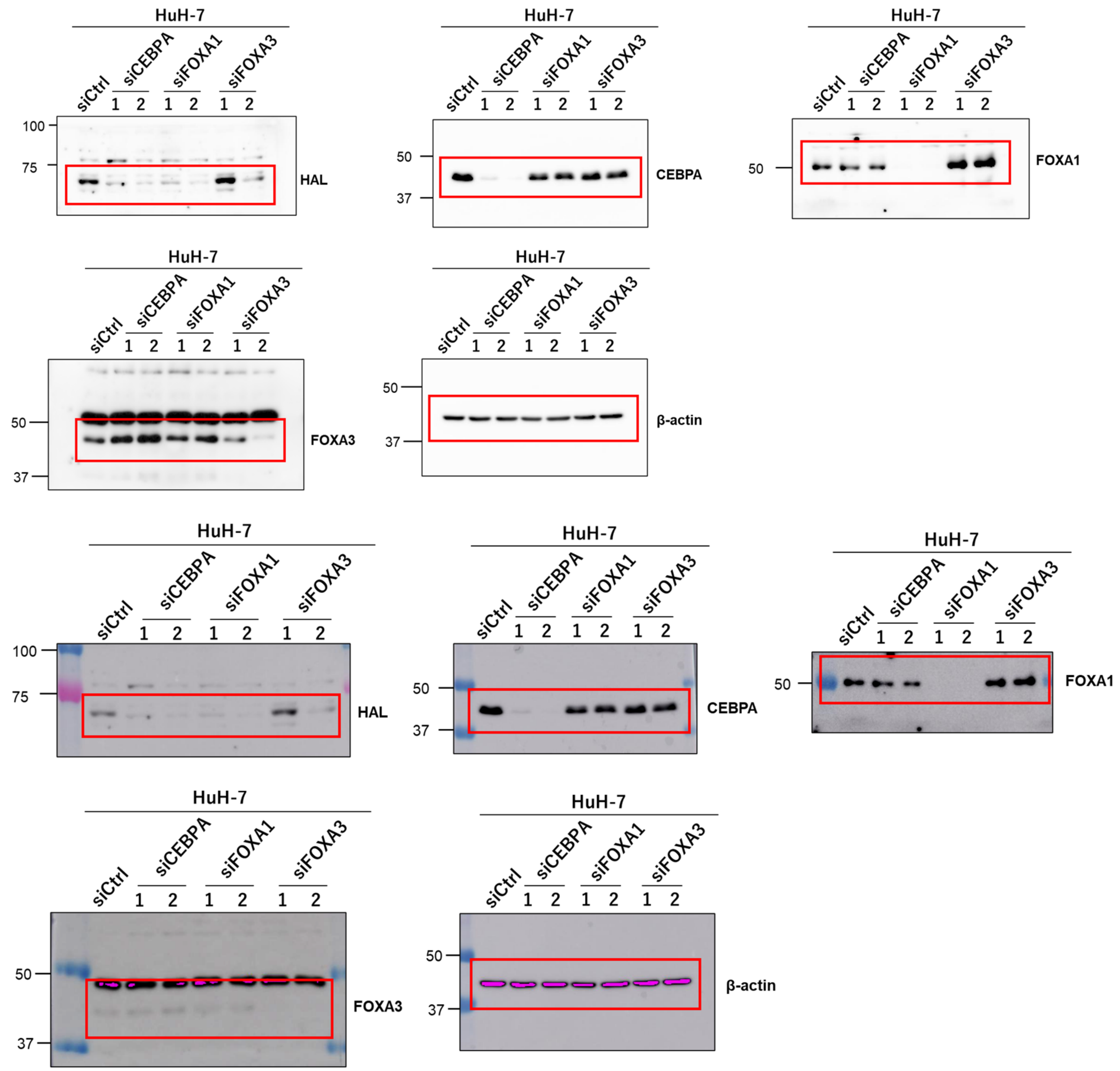

Supplementary Figure 10. Uncropped and unedited blot images with/without ladder of Figure 3b

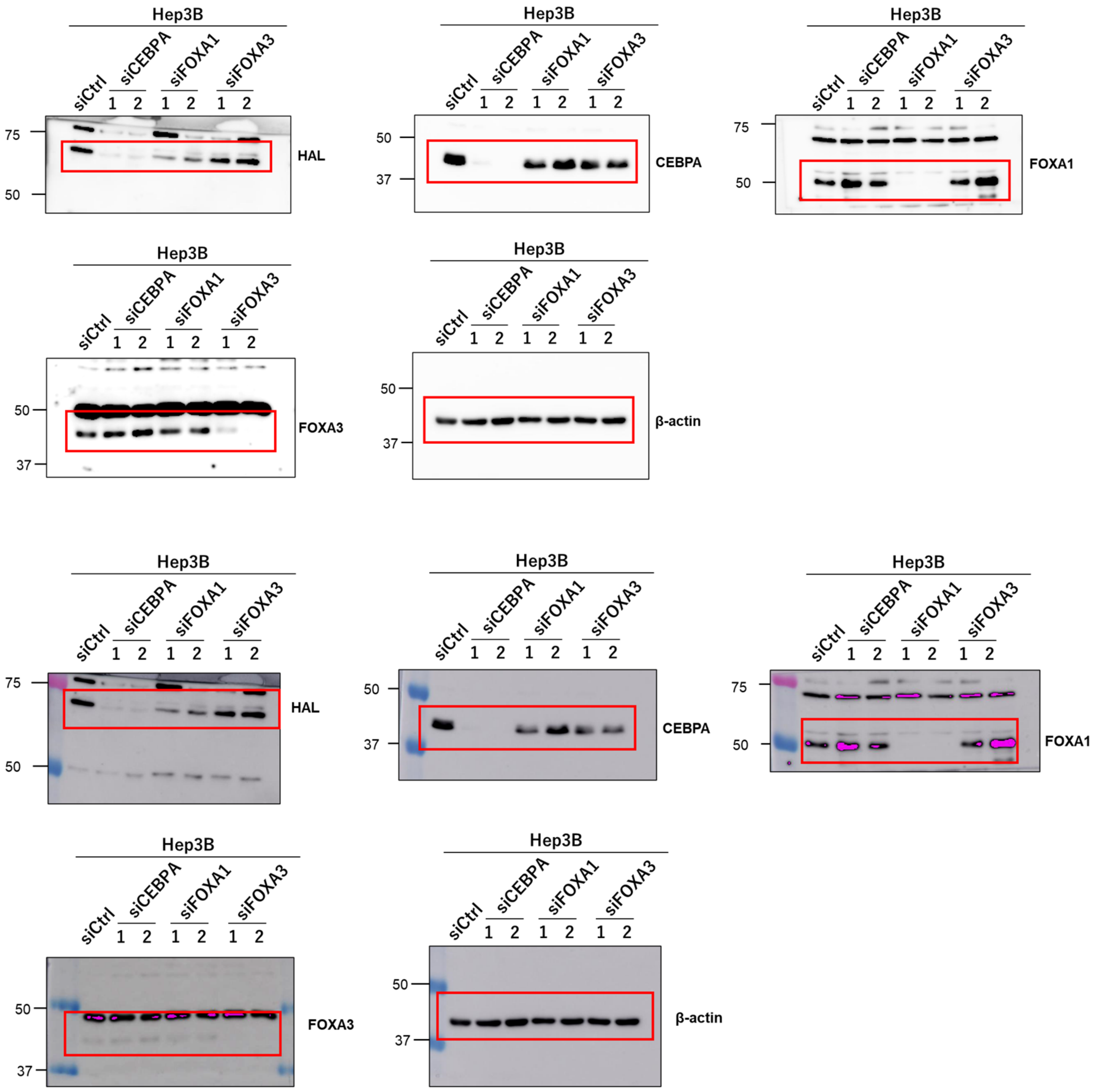

Supplementary Figure 11. Uncropped and unedited blot images with/without ladder of Figure 3b

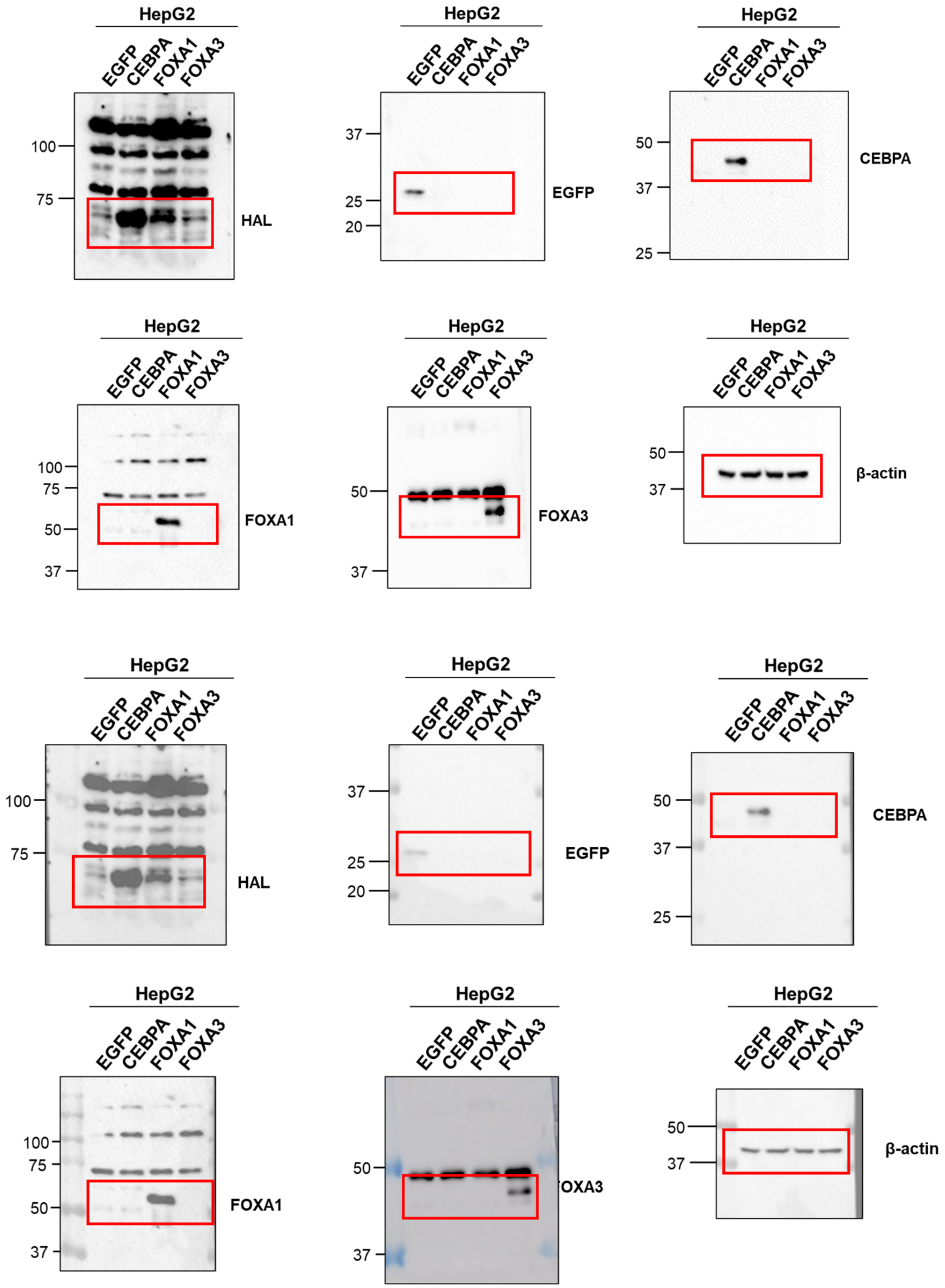

Supplementary Figure 12. Uncropped and unedited blot images with/without ladder of Figure 3d

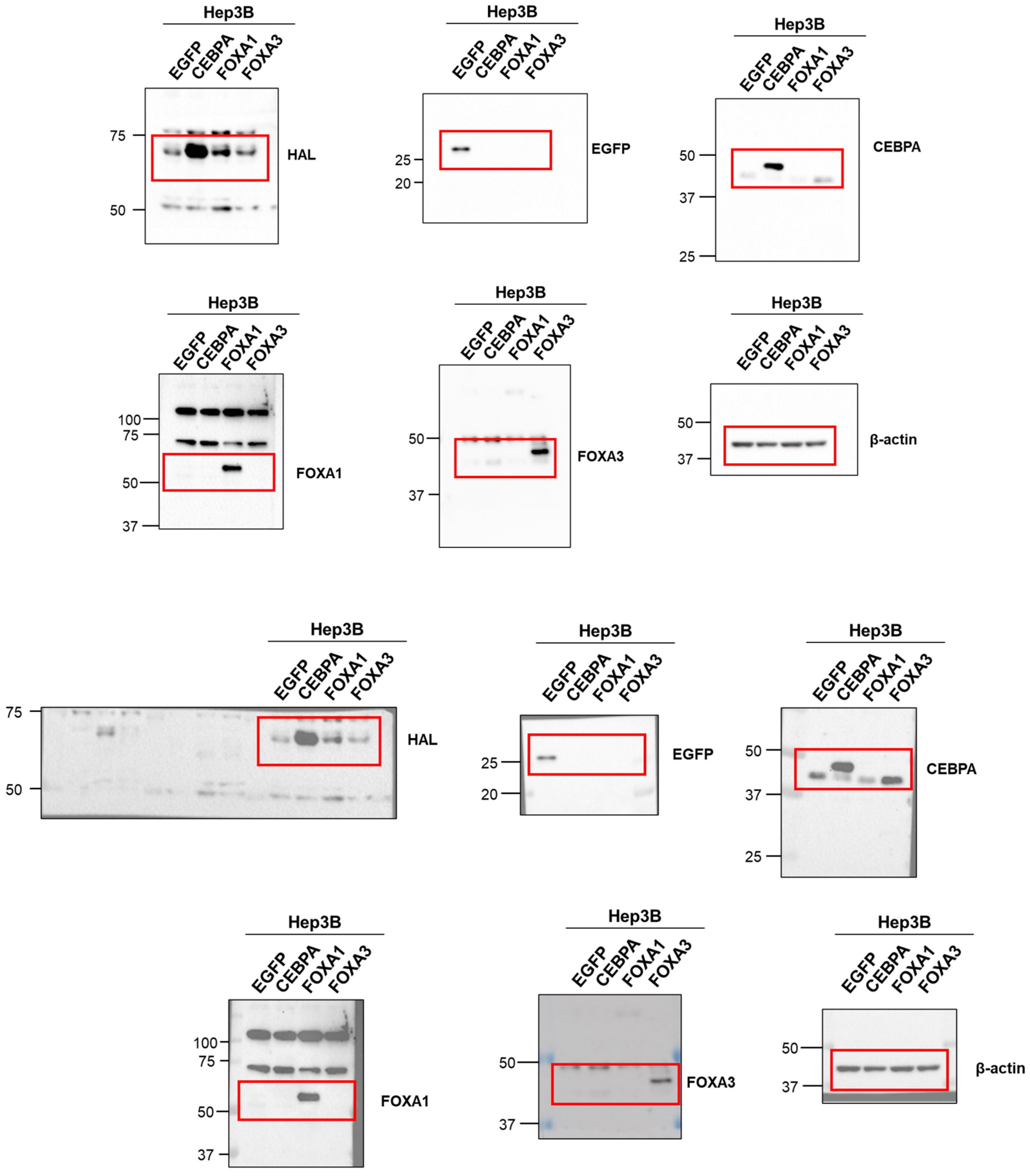

Supplementary Figure 13. Uncropped and unedited blot images with/without ladder of Figure 3d

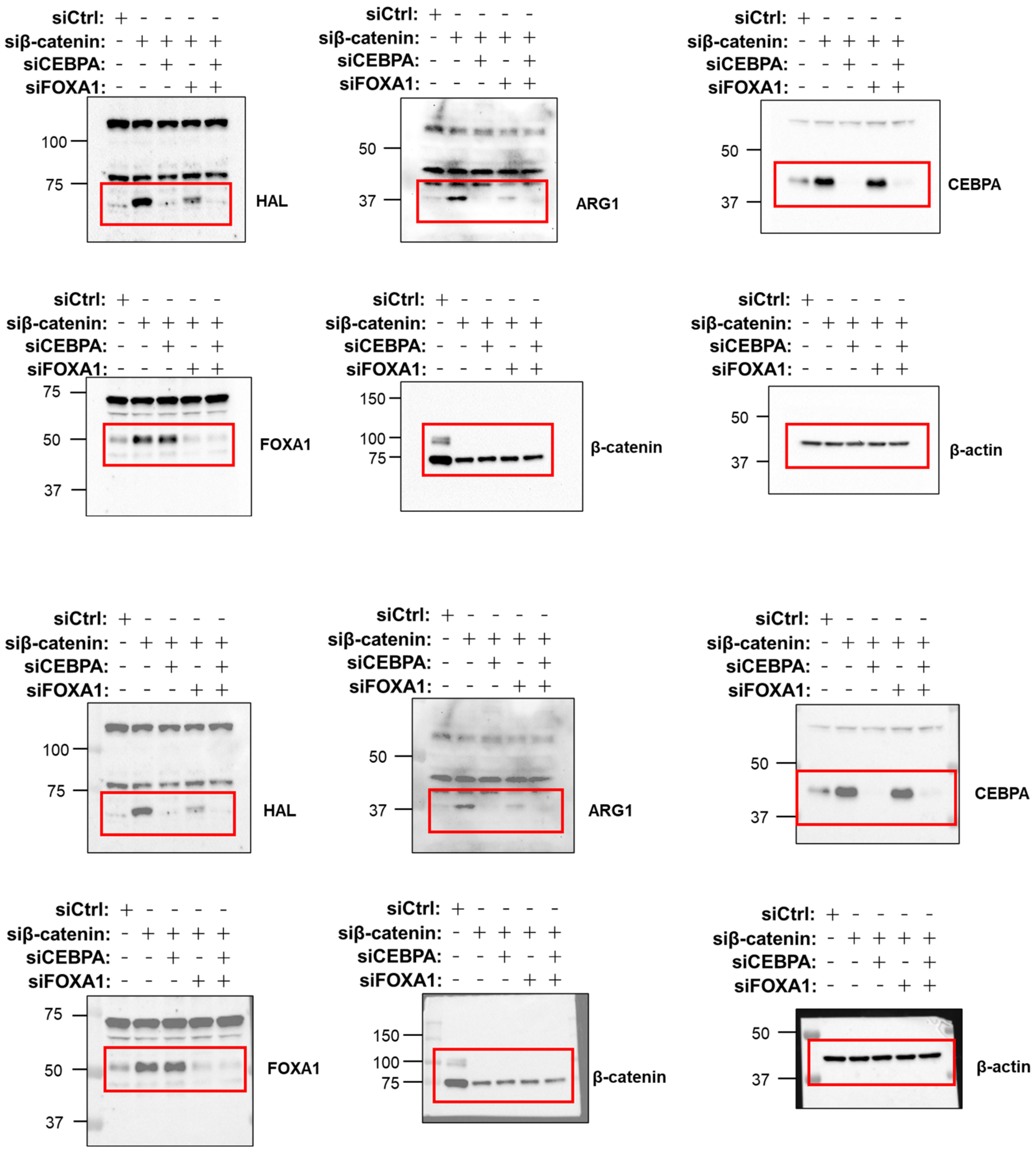

Supplementary Figure 14. Uncropped and unedited blot images with/without ladder of Figure 4h

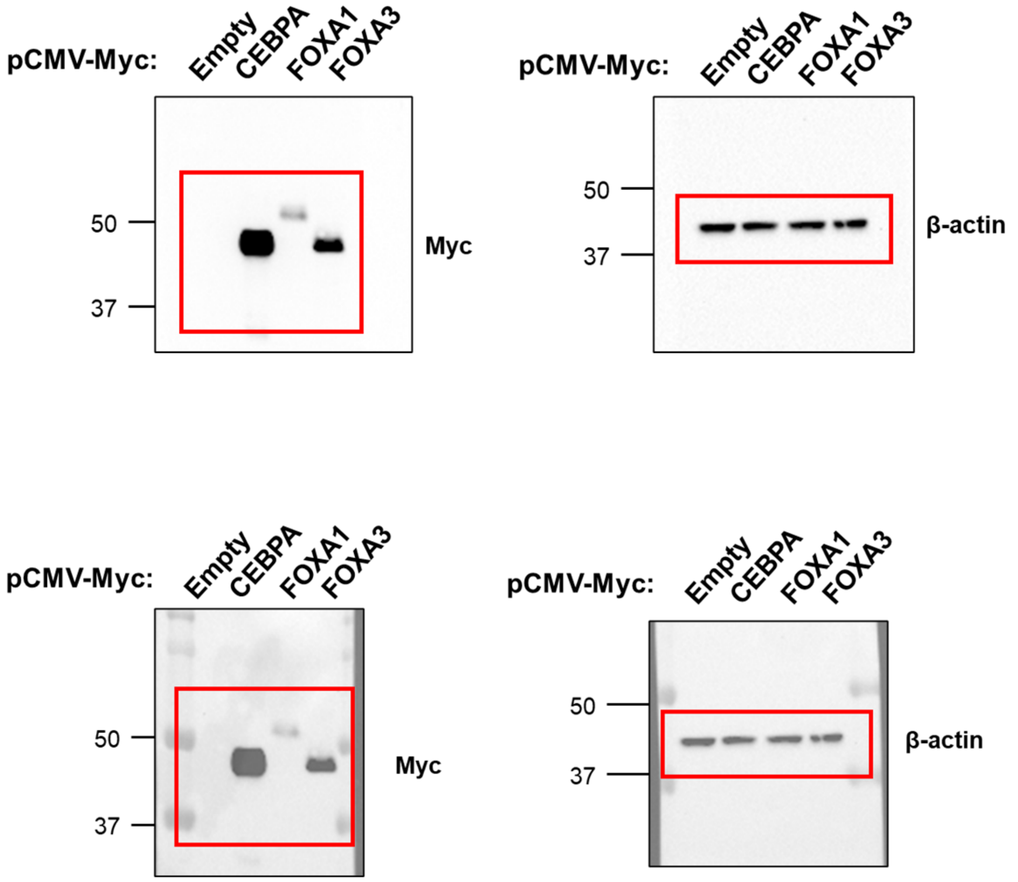

Supplementary Figure 15. Uncropped and unedited blot images with/without ladder of Supplementary Figure 4a

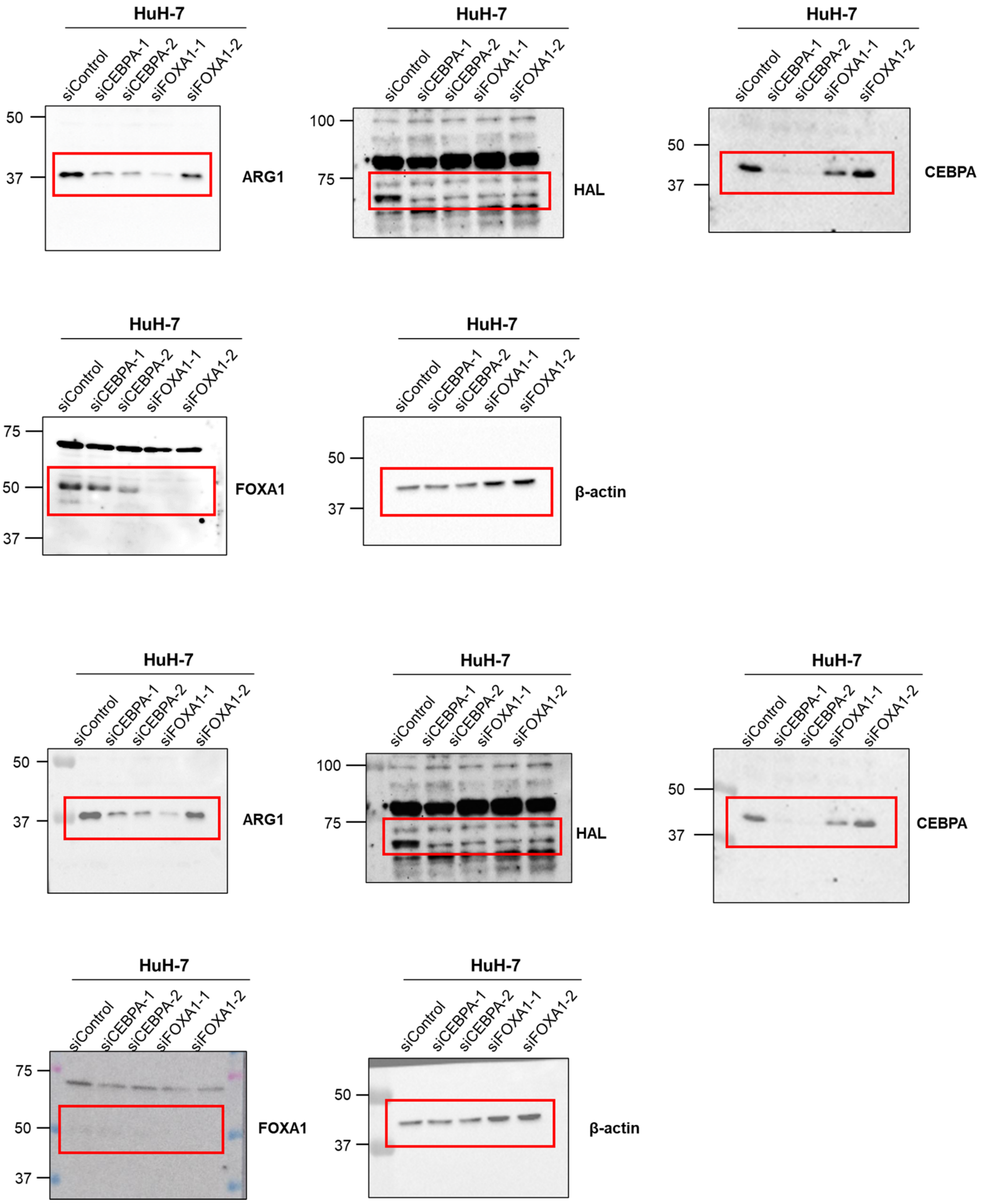

Supplementary Figure 16. Uncropped and unedited blot images with/without ladder of Supplementary Figure 6e
