# Supplementary material for: Wnt/β-catenin signaling regulates amino acid metabolism through the suppression of CEBPA and FOXA1 in liver cancer cells
Source: Commun Biol. 2024 Apr 29;7:510. doi: 10.1038/s42003-024-06202-9 (PMC11058205; doi:10.1038/s42003-024-06202-9)
Supplement: Supplementary file 3 — Description of Additional Supplementary Files [file 42003_2024_6202_MOESM3_ESM.pdf]

Description of Additional Supplementary Files

File name: Supplementary Data 1
Description:
Sheet 1: Data1a. TF binding motifs in the 5'-flanking region of the HAL gene (-90 to -44 bp) predicted by JASPAR
Sheet 2: Data1b. Microarray data from HepG2 cells treated with siRNAs targeting components of β-catenin-TCF/LEF complex
Sheet 3: Data1c. Information of probe IDs corresponding the numbers in Figure 1A

File name: Supplementary Data 2
Description:
Sheet 1: Data2a. Genes significantly altered by CEBPA siRNAs in HuH-7 cells
Sheet 2: Data2b. Genes significantly altered by FOXA1 siRNAs in HuH-7 cells
Sheet 3: Data2c. Significant peaks obtained from the CEBPA ChIP-seq data
Sheet 4: Data2d. Significant peaks obtained from the FOXA1 ChIP-seq data

File name: Supplementary Data 3
Description:
Sheet 1: Data3a. Genes directly regulated by CEBPA
Sheet 2: Data3b. Genes directly regulated by FOXA1
Sheet 3: Data3c. KEGG pathways associated with direct target genes of CEBPA
Sheet 4: Data3d. KEGG pathways associated with direct target genes of FOXA1
Sheet 5: Data3e. Genes directly regulated by both CEBPA and FOXA1
Sheet 6: Data3f. KEGG pathways associated with genes that are regulated by both CEBPA and FOXA1

File name: Supplementary Data 4
Description:
Sheet 1: Data4a. Levels of metabolites in HepG2 cells treated with control, β-catenin, or TCF7L2 siRNA
Sheet 2: Data4b. ORA analysis by MetaboAnalyst

File name: Supplementary Data 5
Description:
Sheet 1: Data5a. Primer sequences used for RT-qPCR, ChIP-qPCR, cloning, and site-directed mutagenesis
Sheet 2: Data5b. Target sequences of siRNAs used in this study

Sheet 3: Data5c. Raw data of Figure 1
Sheet 4: Data5d. Raw data of Figure 2
Sheet 5: Data5e. Raw data of Figure 3
Sheet 6: Data5f. Raw data of Figure 4
Sheet 7: Data5g. Raw data of Figure 5
