# Supplementary material for: Wnt/β-catenin signaling regulates amino acid metabolism through the suppression of CEBPA and FOXA1 in liver cancer cells
Source: Commun Biol. 2024 Apr 29;7:510. doi: 10.1038/s42003-024-06202-9 (PMC11058205; doi:10.1038/s42003-024-06202-9)
Supplement: Supplementary file 9 — Reporting Summary [file 42003_2024_6202_MOESM9_ESM.pdf]

nature portfolio

Corresponding author(s): Kiyoshi Yamaguchi

Last updated by author(s): Apr 1, 2024

Reporting Summary

Nature Portfolio wishes to improve the reproducibility of the work that we publish. This form provides structure for consistency and transparency in reporting. For further information on Nature Portfolio policies, see our Editorial Policies and the Editorial Policy Checklist.

Statistics

For all statistical analyses, confirm that the following items are present in the figure legend, table legend, main text, or Methods section.

n/a	Confirmed	
☐	☒	The exact sample size (n) for each experimental group/condition, given as a discrete number and unit of measurement
☐	☒	A statement on whether measurements were taken from distinct samples or whether the same sample was measured repeatedly
☐	☒	The statistical test(s) used AND whether they are one- or two-sided<br>*Only common tests should be described solely by name; describe more complex techniques in the Methods section.*
☐	☒	A description of all covariates tested
☐	☒	A description of any assumptions or corrections, such as tests of normality and adjustment for multiple comparisons
☐	☒	A full description of the statistical parameters including central tendency (e.g. means) or other basic estimates (e.g. regression coefficient) AND variation (e.g. standard deviation) or associated estimates of uncertainty (e.g. confidence intervals)
☐	☒	For null hypothesis testing, the test statistic (e.g. F, t, r) with confidence intervals, effect sizes, degrees of freedom and P value noted<br>*Give P values as exact values whenever suitable.*
☒	☐	For Bayesian analysis, information on the choice of priors and Markov chain Monte Carlo settings
☐	☒	For hierarchical and complex designs, identification of the appropriate level for tests and full reporting of outcomes
☐	☒	Estimates of effect sizes (e.g. Cohen's d, Pearson's r), indicating how they were calculated

Our web collection on statistics for biologists contains articles on many of the points above.

Software and code

Policy information about availability of computer code

Data collection	N/A
Data analysis	Standard softwares as described in the Methods, including GeneSpring GX14.1, STAR (v2.7.3a), RSEM (v1.3.3), DESeq2 (v1.26.0), Bowtie2 (v2.4.1), MACS2 (v3.6), and HOMER (v4.11).

For manuscripts utilizing custom algorithms or software that are central to the research but not yet described in published literature, software must be made available to editors and reviewers. We strongly encourage code deposition in a community repository (e.g. GitHub). See the Nature Portfolio guidelines for submitting code & software for further information.

Data

Policy information about availability of data

All manuscripts must include a data availability statement. This statement should provide the following information, where applicable:

- Accession codes, unique identifiers, or web links for publicly available datasets
- A description of any restrictions on data availability
- For clinical datasets or third party data, please ensure that the statement adheres to our policy

All data supporting the findings of this study are available within the paper and the supplementary information. Microarray (GSE244527), RNA-seq (GSE244526), and ChIP-seq (GSE244525) data generated in this study were deposited in the Gene Expression Omnibus (GEO) database.

Research involving human participants, their data, or biological material

Policy information about studies with human participants or human data. See also policy information about sex, gender (identity/presentation), and sexual orientation and race, ethnicity and racism.

Reporting on sex and gender	N/A
Reporting on race, ethnicity, or other socially relevant groupings	N/A
Population characteristics	N/A
Recruitment	N/A
Ethics oversight	N/A

Note that full information on the approval of the study protocol must also be provided in the manuscript.

Field-specific reporting

Please select the one below that is the best fit for your research. If you are not sure, read the appropriate sections before making your selection.

☒ Life sciences ☐ Behavioural & social sciences ☐ Ecological, evolutionary & environmental sciences

For a reference copy of the document with all sections, see nature.com/documents/nr-reporting-summary-flat.pdf

Life sciences study design

All studies must disclose on these points even when the disclosure is negative.

Sample size	Sample sizes are given in the figure legends.
Data exclusions	No data are excluded from the experiments and analyses.
Replication	Experiments were repeated as described in the manuscript and in the figure legends.
Randomization	N/A
Blinding	N/A

Reporting for specific materials, systems and methods

We require information from authors about some types of materials, experimental systems and methods used in many studies. Here, indicate whether each material, system or method listed is relevant to your study. If you are not sure if a list item applies to your research, read the appropriate section before selecting a response.

Materials & experimental systems

n/a	Involved in the study	
☐	☒	Antibodies
☐	☒	Eukaryotic cell lines
☒	☐	Palaeontology and archaeology
☒	☐	Animals and other organisms
☒	☐	Clinical data
☒	☐	Dual use research of concern
☒	☐	Plants

Methods

n/a	Involved in the study	
☐	☒	ChIP-seq
☒	☐	Flow cytometry
☒	☐	MRI-based neuroimaging

Antibodies

Antibodies used	All antibodies used are described in the Methods.
Validation	Antibodies were used at the manufacturer's recommended concentrations. Knockdown cell lines were used to query antibody specificity when possible.

Eukaryotic cell lines

Policy information about cell lines and Sex and Gender in Research

Cell line source(s)	HepG2 and Hep3B were obtained from the American Type Culture Collection (Manassas, VA), and HuH-6 and HuH-7 cells were from the Japanese Collection of Research Bioresources Cell Bank (Osaka, Japan). Retrovirus packaging cells PLAT-A was provided from Dr. Toshio Kitamura (The University of Tokyo).
Authentication	All cell lines used were authenticated by short tandem repeat profiling (ATCC and JCRB).
Mycoplasma contamination	All cell lines used were confirmed to be mycoplasma-free by Hoechst DNA staining (ATCC and JCRB).
Commonly misidentified lines (See ICLAC register)	None

Plants

Seed stocks	N/A
Novel plant genotypes	N/A
Authentication	N/A

ChIP-seq

Data deposition

☒ Confirm that both raw and final processed data have been deposited in a public database such as GEO.

☒ Confirm that you have deposited or provided access to graph files (e.g. BED files) for the called peaks.

Data access links *May remain private before publication.*	GSE244525
Files in database submission	All fastq and bigWig files were uploaded.
Genome browser session (e.g. UCSC)	No longer applicable

Methodology

Replicates	A ChIP-seq experiment with single technical replicate was performed, and the significant peaks were further confirmed by ChIP-qPCR.
Sequencing depth	58.6, 61.6, and 67.3 million reads were obtained from the input, CEBPA, and FOXA1 samples, respectively.
Antibodies	Antibodies used for ChIP-seq are described in the Methods.
Peak calling parameters	Peak calling was performed using MACS2 (v3.6). Peaks with q-value < 0.05 were considered significant.
Data quality	A total of 4,460 and 7,469 signficant peaks were obtained from the CEBPA and FOXA1 ChIP-seq samples, respectively (q-value < 0.05).
Software	The sequencing data were aligned to human genome (GRCh38) using Bowtie2 (v2.4.1). Peak calling followed by assignation of peaks to genes was performed using MACS2 (v3.6) and HOMER (v4.11).
